# Preliminary Study of Microelements, Phenolics as well as Antioxidant Activity in Local, Homemade Wines from North-East Greece

**DOI:** 10.3390/foods9111607

**Published:** 2020-11-05

**Authors:** Adriana Skendi, Maria Papageorgiou, Stefanos Stefanou

**Affiliations:** 1 Department of Agricultural Biotechnology and Enology, International Hellenic University, 1st Km Dramas-Mikroxoriou, GR-66100 Drama, Greece; 2 Department of Food Science and Technology, International Hellenic University, POB 141, GR-57400 Thessaloniki, Greece; mariapapage@food.teithe.gr; 3 Department of Agriculture, International Hellenic University, POB 141, GR-57400 Thessaloniki, Greece; stefst2@ihu.gr

**Keywords:** antioxidant activity, heavy metals, ICP-OES (inductively coupled plasma-optical emission spectrometry), macroelements, microelements, total phenolic content, wine

## Abstract

The present study is aimed to analyze the composition of microelements in wines prepared by amateur winemakers in Drama region (recognized as Protected Geographical Indication (PGI)), especially the toxic ones, as well as the phenolic content and antioxidant activity of these wines since moderate wine consumption may contribute to health benefits to the consumers. A total of twenty-four wines, comprising sixteen red and eight white, were tested. The micro and macroelements were determined with ICP-OES (Inductively coupled plasma-optical emission spectrometry). Chromatic characteristics of wines and total phenolic and flavonoid compounds as well as their antioxidant activity with three different assays were determined spectrophotometrically. The amateur’s wines showed levels of contaminants with toxic metals less than the limits set from the International Organization of Vine and Wine (OIV). Moreover, toxic metals concentrations were comparable to those of European wines. The Principal Component Analysis discriminated wines into white and red and further distinguished the red wine into two groups depending on the Total Flavonoid content, antioxidant activity and color intensity. The moderate consumption of amateur wines can be recommended since their consumption is not harmful to the health, and they contain high amount of phenolics and flavonoids comparable or even higher to that of commercial wines.

## 1. Introduction

The consumption of wine provides a great number of essential elements to the human organism. Phenolics are secondary metabolites present in vines and vary in nature and level. Presence of phenolics in wine is strongly affected by several factors such as grape variety and location, cultivation practices, ripening stage at harvesting time and vinification techniques [1,2]. They are considered responsible for the color and taste of the produced wine as well as the positive effects on human health (i.e., antioxidant, anti-inflammatory, antimicrobial) [3]. Phenolic compounds due to their antioxidant activity influence the creation or the loss of components responsible for the aroma and the taste of wines as well as for the modification of wine color during ageing.

On the other hand, the presence of redox metals could also boost these mechanisms. This is because ions of copper, iron and manganese form stable complexes with phenolic compounds and amino acids during wine maturation on storage that in their turn are responsible for the color, aroma and taste of wine. High levels of K, Ca, Cu and Na can be associated with mineral levels in the soil, fertilization and pesticide treatment practices or fining and clarifying substances added to wine [4,5]. In the literature, Cu levels vary from 0.020 to 2.60, Fe 0.06–23.7, Mn 0.1–5.5 and Zn 0.02–4.63 mg/L among the international wines [5,6]. In Greek wines, the levels of Cu, Fe, Mn and Zn are 0.2–0.9, 1.1–5.6, and –2.3 and 0.3–8.9 mg/L, respectively [7].

Among the elements present in wine, heavy metals, such as arsenic, lead and cadmium, can be found that are considered dangerous at high levels since several health issues can arise from the exposure to them. Heavy metals pass into alcoholic beverages from the raw materials as well as during different stages of processing through equipment and package used for their ageing and/on storage. Contamination and their accumulation in wine may happen during the contact with machinery, containers, pipes (stainless steel, brass, aluminum, wood) and even filter aids [6,8,9,10]. Among the heavy metals, Pb and Cd are considered as the most toxic elements with maximum allowable limits of 0.20 mg/kg wet weight set up for Pb from EC [11] and 0.01 mg/L for Cd, recommended from International Organization of Vine and Wine (OIV) [12]. The literature reports lower mean Pb values for the United States red wines (4.4 μg/L) compared to international red and white wines (mean values 33.9 μg/L and 35.7 μg/L, respectively) [13]. Although recognized as an essential element, copper at levels higher than 1 mg/L, is considered toxic [12]. Besides the toxicity, high levels enhance wine oxidation, especially visible as browning in white wine, and contribute to haze formation [14]. Generally, fining treatments that are performed in order to clarify and stabilize wines (by removing unwanted compounds that impair sensorial characteristics) are based on materials that bind the target compounds forming insoluble aggregates that precipitate. On the other hand, it was observed that the use of fining agents can also affect the elemental profile and color of the wines [15,16].

In different areas of many countries, the so-called homemade wine still dominates on the table at important family rituals. In Greece, the number of amateur producers that make wine in small quantities, mainly for own consumption, is very high. It is roughly estimated that in Greece, the bulk wine sales reach 60% of the total wine production. Although a high percentage of the bulk wine comes mostly from small and medium-sized production units or cooperatives, amateur winemakers cover a significant part that must be taken into account. Bulk wine produced does not bear an identity and no one can certify its quality. There exists the conviction that amateur producers produce poor quality wine due to lack of knowledge and education. Beside using the produced wine for personal consumption, they share it with relatives and friends as well they may sell it to the local markets at extremely low prices. The region of Drama represents the home to some of the finest wineries in Greece and is recognized since 1995 as Protected Geographical Indication (PGI). Located in the northeast part of the region of Macedonia in Greece (mainly Mediterranean climate), it offers ideal microclimates for many international and local grape varieties. According to the statistical data obtained from the Hellenic Ministry of Rural Development and Food, in 2018 the vineyards covered an area of 616.1 ha in the Drama region with a grape production of 6516 tones [17].

Although determined in commercial wines [18], to date, the antioxidant activity of wines produced by amateur winemakers in Greece has not been estimated. In general, there is missing information regarding wines produced by amateur winemakers in Greece. The lack of available information impedes wine producers to take advantage of the value associated with the antioxidant activity of wine or to take the appropriate measures to decrease the presence of heavy metals by changing winemaking techniques in order to increase the antioxidant capacity of their wines.

Besides the grape variety or the vintage year, the procedures applied by the wineries affect the chemical composition of wine. Few, if any, of the homemade wines are analyzed for the micro and macro elements as well as their phenolic content and antioxidant activity. It is of interest to compare the micro and macro element content of homemade wines made from locally growing vines since they play an important role in the local wine market. Moreover, no report exists on toxic metals presence in these local home-made wines.

Taking into consideration all the above, the aim of the present study was to determine the elemental composition and assess the phenolic content as well as the antioxidant capacity of wines produced in Drama region. Another objective was to determine components that could differentiate amateur wines. To this end, exploratory studies were carried out through Principal Component Analysis (PCA) while Hierarchical Cluster Analysis (HCA) was performed to visualize differences/similarities between wines of the region.

## 2. Materials and Methods

### 2.1. Reagents

Methanol and water used was of HPLC (High-Performance Liquid Chromatography) gradient grade and was purchased from Chem-Lab NV (Zedelgem, Belgium). There were used single element standard solutions 1000 mg/L, of As, Cd, Cr, Cu, Fe, Mn, Ni, Pb, Zn, K, Na, Ca and Mg (Sigma Chem, St. Louis, MO, USA). For trace metal analysis, all glassware and plastic containers used were washed properly, first with nitric acid and then with ultra-pure water, in order to ensure that any contamination does not occur. All the other chemicals and reagents were of analytical grade.

The DPPH (2,2-diphenyl-1-picrylhydrazyl) and (+)-catechin were from Sigma Aldrich, St. Louis, MO, USA, whereas TPTZ (2,4,6-tripyridyl-s-triazine) and aluminum chloride-6-hydrate were obtained from Alfa Aesar, GmbH&GoKG, Karlsruhe, Germany. ABTS (2,2’-azinobis (3-ethylbenzothiazoline-6-sulfonic acid), Trolox ((S)-(-)-6-hydroxy-2,5,7,8- tetramethylchroman-2-carboxylic acid), and gallic acid were from J&K Scientific GmbH, Pforzheim, Germany. Folin-Ciocalteu reagent, sodium acetate trihydrate and sodium hydroxide (HPLC grade) were obtained from Chem-Lab NV, Zedelgem, Belgium. Iron (III) chloride hexahydrate, sodium carbonate, and sodium nitrite was from Merck, KGaA, Darmstadt, Germany. All the other chemicals used were of analytical grade.

### 2.2. Sample Collection

A total of 24 samples of wine of which 8 white and 16 red were collected by amateur winemakers of Drama region (Coordinates: 41°15′ N 24°10′ E, Total area 3468 km^2^) in Greece presenting their products in the Tasting Competition of Amateur Wines and Spirits hold in the region of Drama, Greece, in 2017 and considered of bearing high sensorial attributes. All wines were produced for home-use and were non-officially marketable. Sampling was done at random from students of the Department of Oenology and Beverage Technology in the Association of amateur winegrowers/winemakers of Drama, Macedonia, Greece. Wines were produced with grapes from different cultivars and were of the vintage of 2011–2016. Their coded names are reported in Table 1. These wines were presented in plastic or glass bottles and produced according to the practices followed by the individual winemaker.

The obtained samples were stored in the dark and low temperature. Aliquots from the stored samples were poured in glass tubes tightly sealed with Teflon liner caps and stored at 4 °C till analysis. The glass tubes were previously washed with nitric acid and then with water in order to avoid cross-contamination.

### 2.3. Determination of Heavy Metals and Trace Elements

Two different subsamples were taken from each wine. Samples were diluted with ultra-pure water that had been acidified with 8% nitric acid in order to minimize the signal suppressing effects of the organic constituents of wines, without losing the ability to detect elements present at ppb concentration range. Simple acidic dilution of wines gives similar results when tested with ICP-OES with sample mineralization [19]. The original solutions of extracts were 1:2 (*v/v*) diluted to measure the As, Pb, Cd, Zn, Ni, Cr, Fe, Mn and Cu content and 1:10 (*v/v*) diluted to measure macro-elements K, Na, Ca and Mg content. All the obtained samples were analyzed three times on a Perkin--Elmer 8300 DV (ICP-OES) Inductively Coupled Plasma Optical Emission Spectrometer (Perkin-Elmer, Waltham, MA, USA).

Operating conditions comprise nebulizer flow 0.8 L/min, auxiliary gas flow 15 L/min, sample uptake rate 1.50 mL/min, plasma power 1300 W and integration time 15 s. Measurements were performed in axial view for As (188.979), Pb (220.353), Cd (228.802), Zn (206.200), Ni (231.604), Cr (267.716), Fe (238.204), Mn (257.610), Cu (327.393), K (766.490), Na (589.592), Ca (317.933) and Mg (285.213) with the numbers in brackets showing the wavelength in nm that was used for each element. The method for the determination of the abovementioned metals was developed according to the IUPAC guidelines [20].

Calibration was performed using stock solutions of the metal ions appropriately prepared from the standard solutions that were purchased. Standard stock solutions containing each compound were diluted in the appropriate amounts with ultrapure water to obtain working calibration standard solutions. The calibration blank consisted of ultrapure water acidified with nitric acid. In general, there were three replicate injections performed to obtain the linear regression values from the average absorbance of each analyte. Very good linearity (R^2^ > 0.9999) of calibration curves was observed for all analytes tested. Coefficient of variation (CV) for each studied compound was calculated as the ratio of the standard deviation to the average value at different levels of concentration in the linear range. Moreover, the limit of detection (LOD) and limit of the quantification (LOQ) of the method have been calculated as recommended by IUPAC [20]. For the calculation of LOD and LOQ, we used the values obtained from the standard deviation of the intercept (SD) and the slope of the calibration curve (S) as deduced of mathematical expressions: LOD = 3.3 SD/S and LOQ = 3 × LOD. Recovery study revealed that the method applied was satisfactory showing values in the range of 87.5–104%. Moreover, the method resulted in satisfactory repeatability (RSD < 10%). The detection limit was 4.2, 1.4, 2.7, 0.9, 4.6, 9.7, 2.6, 2.8, 5.9, 63.1, 15.0, 79.3 and 10.0 μg/L for As, Cd, Pb, Cr, Cu, Fe, Mn, Ni, Zn, K, Na, Ca and Mg, respectively. Each sample was tested in duplicate, and three separate analyses were made for each sample. Moreover, a blank was run for every set of ten samples to check for interferences or contamination.

### 2.4. Determination of Colour

The wines were characterized based on their chromatic properties. The red wines are assessed by measurements at three wavelengths, 420, 520 and 620 nm. Intensity (I) and hue (T) of the color of red wines were determined by the method of Sudraud [21] modified by Glories [22] and measured in a GENESYS™ 10S UV–Vis (Ultraviolet-Visible) Spectrophotometer (Thermo Scientific, Swinton, UK). The above-mentioned parameters were calculated as follows:

The intensity (I) = A_420nm_ + A_520nm_ + A_620nm_, where A_420nm_, A_520nm_ and A_620nm_ represent the yellow, red, and blue components of the color intensity.

Hue (T) = A_420nm_/A_520nm_

Moreover, the color composition was calculated as the percentage that each of the three components of wine intensity has in the color of the wine as well as the brilliance of red wines:

Yellow: A_420_ (%) = (A_420_/I) × 100; Red: A_520_ (%) = (A_520_/I) × 100; Blue: A_620_ (%) = (A_620_/I) × 100; Brilliance (%) = [1 − [(A_420_ + A_620_)/2 × A_520_]] ×100

Before performing the measurements, all wine samples were centrifuged for 10 min at 4000 rpm to remove any particles that could interfere with spectrometric measurements.

Measurement of white wines at 280 and 420 nm wavelengths was selected as these wavelengths are commonly used for monitoring the “total phenolic index” and” Browning” in white wines, respectively [23,24,25].

### 2.5. Determination of Total Phenolic Content and Total Flavonoid Content

For the TPC (total phenolic content) and TFC (total flavonoid content) measurements the wine samples were centrifuged (4000 rpm for 15 min) and the supernatants were collected and measured. TPC was determined using the modified Folin Ciocalteu’s method [26], using gallic acid as a standard. Briefly, to 200 µL of wine were added first 0.8 mL of Folin–Ciocalteu reagent (diluted with distilled water 1:10 (*v/v*) and allowed to react for 2 min before adding 2 mL of 7.5% *w/v* sodium carbonate aqueous solution. Then the volume of mixture brought to 10 mL with distilled water. After 60 min of storage in the dark, the absorbance was read at 750 nm, and the results were expressed as milligrams of gallic acid equivalents per one mL sample (mg GAE/mL).

TFC was determined following the modified method of Bao, et al. [27] using catechin as a standard flavonoid. An aliquot of 300 μL of wine sample was mixed first with 225 μL NaNO_2_ (5% *w/v*), vortexed and allowed to react for 5 min before adding 225 μL AlCl_3_ × 6H_2_O (10% *w/v*), again vortexed and allowed to react for another 5 min. Finally, it was added 750 μL 1M NaOH and allowed to develop color in the dark for 30 min before measuring the absorption at 510 nm. The results were expressed as milligrams of catechin equivalents per one mL sample (mg CATE/mL). For both, TPC and TFC, the measurements were performed at least in triplicate.

### 2.6. Determination of Antioxidant Activity

The antioxidant potential of wines was determined with DPPH, ABTS and FRAP (Ferric reducing antioxidant power) assays using Trolox as antioxidant standard as previously reported in Skendi, et al. [28].

In order to determine ABTS antioxidant activity, first, ABTS^•+^ stock solution was prepared by mixing 0.108 g of the ABTS salt with 0.0198 g potassium persulfate in 100 mL of distilled water. The solution was allowed to stand in the dark for at least 16 h before its use. Just before measurements, the ABTS^•+^ stock solution was diluted with distilled water until reaching an absorbance value of 0.700 ± 0.020 at 734 nm. Each wine sample (100 µL) was mixed with 3.9 mL of the freshly prepared ABTS^•+^ solution, and absorbance was measured at 734 nm after 4 min of storage in dark at room temperature.

The ability of wines samples to scavenge DPPH free radicals was determined by mixing an aliquot (150 µL) of wine sample with 2.85 mL of freshly prepared DPPH^•^ (100 µM). The samples were allowed to react for 5 min in dark and at room temperature before measuring the absorbance at 516 nm.

The ferric reducing/antioxidant power (FRAP) of wine samples was measured by mixing 100 µL of wine sample with 3 mL of working FRAP solution daily prepared. The mixture was allowed to react for 4 min at 37 °C and the absorbance measured at 593 nm against a blank. The FRAP solution consisted of a mixture of three solutions: 20 mM ferric chloride solution, 10 mM TPTZ solution in 40 mM HCl, and 0.3 mM (pH 3.6) acetate buffer in a proportion of 1:1:10 (*v/v/v*), respectively.

Results determining the antioxidant activity (ABTS, DPPH and FRAP) were expressed as mg Trolox equivalents per mL of wine sample (mg TE/mL).

### 2.7. Statistical Analysis

In order to provide new insights into the composition of the wines made by amateur winemakers, several statistical analyses were used. Non-parametric test for independent-samples median test was performed using SPSS 25 package. Kruskal–Wallis test was used to detect evidence if red and white wine samples with similar distribution have the same median (*p* < 0.05). Samples with concentration below the LOD were assigned a value of 0 concentration. Pearson linear and Spearman’s correlation (2-tailed, *p* < 0.05) were employed to recognize any relationship between parameters studied depending on the type of data distribution. The SPSS Statistics 25.0 software (SPSS Inc., Chicago, IL, USA) was used to perform these tests in wines.

The mathematical processing of the standardized data for wine was done by hierarchical cluster analysis (HCA) using Ward’s method. The results from the cluster analysis are presented by a dendrogram. Moreover, Principal Component Analysis, PCA, using Pairwise estimation method, was carried out on the data obtained from wines in order to classify them according to several factors: type of wine (red, white), vintage, color characteristics, TPC, TFC and antioxidant activity. HCA and PCA analyses were performed using JMP 14 (SAS Institute Inc., Cary, NC, USA).

## 3. Results

### 3.1. Metals in Organic Wine Samples

The metal content of the wines as well as the limits set by the OIV [12] for the presence of toxic metals in wines are presented in Table 2. The amount and the elemental profile of wines depend on the type of soil, the capacity of the grapes to absorb the various metal ions and the winemaking procedures [29]. It is of great importance to monitor the levels of toxic metals in wines prepared by amateur winemakers since moderate wine consumption may contribute to the daily intake of these metals [30].

Arsenic was not found in any of the studied wine samples suggesting that arsenate pesticides were not used for grape production. Similarly, in another study performed in Greece, Cretan commercial wines were free of arsenic [7].

Cadmium, lead and chromium levels determined in all analyzed wines were clearly below (0.005, 0.067 and 0.020 mg/L, respectively) the lowest threshold limit set by the OIV [12] (0.01, 0.1 and 0.1 mg/L, respectively). Kruskal–Wallis test on all the samples revealed that the type of wine plays a significant effect on the content of these three elements (Cd, Pb and Cr). All the three elements were found in higher concentration in red wines when compared to the white counterparts.

In a study of 150 German wines, the Cd levels ranged from 0.003 to 0.98 μg/L [31] being lower than those observed in the present study (1.56 to 4.86 μg/L). In two studies performed in commercial Greek wines dating back to 1989 and 2002, the amount of Pb varied from 0.20 to 0.41 mg/L and from 0 to 0.0065 mg/L [7], respectively, suggesting an improvement in the cross contamination effect over time. Contrary, a more recent study, dated 2019, performed in commercial wines produced both from professionals and amateur winemakers, reports higher amounts of Pb ranging from 0.005 to 0.284 mg/L and from 0.009 to 0.325 mg/L [32], respectively, surpassing the limits set by OIV. Two of their samples, originated also from Drama region, showed values of 0.112 and 0.263 mg/L for the professional and amateur sample, respectively, both higher than the limits. The authors attributed their findings to the low-quality galvanization of the equipment used. Moreover, Pb values in our study were much lower than those reported for European wines by Banović, et al. [33] (0.067–0.355 mg/L in Croatian red wines), Ostapczuk, Eschnauer and Scollary [31] (0.004–0.254 mg/L German wines) and Mena, et al. [34] (0–1.125 mg/L in Spanish wines). In a recent study about Italian wines, the mean amount of lead in the 27 red wines (17.164 µg/kg wet weight) is higher than in the 23 white (10.068 µg/kg wet weight) wines studied. The maximal values reported in the same study are 43.816 µg/kg wet weight and 26.232 µg/kg wet weight in red and white wines, respectively [35]. Suggested that bentonite has the ability to remove part of the Pb present in wine.

Chromium values in the studied wine samples varied from less than the limit of detection to 0.020 mg/L, lower than the limit set by OIV (0.1 mg/L) [12]. These values are lower than those reported for white 0.01–0.26 mg/Land red 0.01–0.41 mg/L Greek wines [36]. The amount of nickel in the wines varied from 0.055 to 0.357 mg/L for red and from 0.061 to 0.486 mg/L for white wines, the findings being higher that the values reported for French white (0.0075–0.0745 µg/L) and red wines (0.0054 to 0.0879 µg/L) [37] and those present in commercial Greek white (0.00–0.13 mg/L) and red (0.00–0.10 mg/L) wines [36]. The use of nickel in alloys of stainless steel could increase the level of Ni in wine. Stainless steel used for wine tanks contains both chromium and nickel. A positive significant correlation (0.421 at the 0.05 level, 2-tailed according to Spearman’s Correlation) was observed between the presence of these two elements suggesting that possibly part of the amount of these two compounds in wines may be a result of corrosion on stainless steel equipment possibly due to the SO_2_ that facilitates the extraction of these compounds from the steel alloy fermentation vessels.

Zinc was found in all analyzed wine samples at concentration levels lower than 1.031 mg/L for red wines and 1.210 mg/L for white wines, being less than the legislative limit (5 mg/L). The difference observed between the red and white wines is statistically significant at the level 0.05. Values of zinc reported for commercial Greek wines varied from 0.05 to 1.80 for white and from 0.05 to 1.40 mg/L for red wines in one study whereas in another study values, regardless the type of wine, varied from 0.06 to 5.5 mg/L [7]. In Croatian red wines, the levels ranged from 0.266 to 2.434 mg/L [33]. Zinc is naturally present in grapes in small quantities whereas higher quantities are linked with the use of zinc containers (tin plated equipment) for the production and the storage of the wine or from pesticides.

In the present study, the concentration of iron varies from 0.309 to 1.672 mg/L being much lower than the limit of 10 mg/L that could cause instability of the wines. These values are also lower than the values reported in other Greek and Croatian [33] wines. No significant differences were observed among the white and red wines values of iron.

Copper levels observed in our samples were less than 0.206 mg/L for the white wines and 0.144 mg/L for the red wines without this difference being statistically significant. These values are clearly lower than the legal limit of 1 mg/L set by the OIV that causes turbidity in wines. Moreover, these values also differ from those reported for commercial Greek wines. In specific, they are lower than those reported from for white (0.01–1.65 mg/L) and red (0.01–1.00 mg/L) wines and lower than the lower level reported for amateur wines (0.243–0.792 mg/L) from Papageorgiou, Karampatea, Mitropoulos and Kyzas [32]. Copper at low doses is involved in oxidative reactions that take place during wine ageing, promotes oxidation of iron and white casse. In addition, when present at concentrations around 1 mg/L, it causes turbidity. Our findings are lower than the values (range 0.050–0.394 mg/L) reported for German wines and Croatian wines (range 0.212–1.23 mg/L) [33]. These very low levels of Cu and Fe observed in our samples may be related to the increased amount of bentonite used as well as the process of winemaking applied, i.e., wine exposure to oxygen at any stage of vinification [15,38,39]. It was observed that the binding capacity of Cu and Zn to either bentonite and insoluble wine matrix compounds (lees) increases in wines exposed to oxygen, resulting in the removal of these metals from the wine supernatant [39]. The fact that Ca and Mg were negatively correlated with Cu (r = −0.550, at *p* < 0.1)) and Zn (r = −0.407, at *p* < 0.05), respectively, suggests that the release of Ca and Mg from bentonite is associated with the binding of Zn and Cu. 

The amount of Mn in wines was found to be lower than 4.667 mg/L for red, and 0.673 mg/L for the white wines with this difference being statistically significant. The values observed for red wines are higher than those reported for both Cretan wines (up to 2.2 mg/L) -although they report an exception of 10 mg/L [7] and Spanish wines [40]. Catarino, Madeira, Monteiro, Rocha, Curvelo-Garcia and de Sousa [15] reported that the Mn could be increased in wines due to the bentonite used.

The macroelements K, Na, Ca and Mg are found at concentrations lower than 123.2, 5.3, 8.8 and 12.1 mg/L. These values are less than those reported in the literature for these elements for Greek red and white wines [36] and Spanish wines [40]. Although high K concentrations are typical for red wines since they are involved in the equilibrium of red color pigments complex with anthocyanin and tartaric acid, in the present study there was not observed significant difference in the levels of K between the two types of wine. Moreover, red and white wines studied did not differ in their content of Na, Ca and Mg.

### 3.2. Colour of Wines

Color is considered as one of the principal attributes of wine and it is recognized decisive for the consumer’s preferences. The variation in color can help in the recognition of the typical characteristics of a wine and can be attributed to the influence of the climatic as well as vinification procedures. Color stability during the process of vinification and ageing are considered reliable indicators to monitor wine quality. Color of red wine is generally determined spectroscopically measuring the contribution of red, yellow and blue components at 520, 420 and 620 nm, respectively. With ageing, the value at 520 nm decreases, whereas those measured in 420 nm and 620 nm increase due to the development of the structure of anthocyanins [41]. Data from chromatic parameters of examined red and white wines are presented in Table 3.

Wine color intensity (I) varies greatly in the different types of the wines studied (Table 3). The studied wines can be characterized as light (8) and medium (8 samples) body wines according to Iland, Ewart, Sitters, Markides and Bruer [42]. It is recognized that the color intensity depends on pigment composition and state of pigment equilibrium of the wine, but factors such as pH and SO_2_ could affect it [43]. Oxygen exposure during the winemaking process may result in wines with lower concentrations of anthocyanins and tannins and color measures that reassemble the characteristics of aged wines [38].

Wine hue (T) is a measure of wine tint and indicates the development of color towards orange during ageing with young wines showing values <1 (0.5–0.7), and aged wines reaching an upper limit of around 1.2–1.3. During red wine maturation, wine color hue changes are observed through a decrease in absorbance at 520 nm and an increase in absorbance at 420 nm [44]. The red wines exhibited hue values of 0.655 to 1.176. Six of the samples showed values higher than one and are considered aged. With ageing, the color intensity of wines slightly decreases whereas the hue slightly increases [41]. The literature reports that the hue is affected by the winemaking procedures and shows higher values in the O_2_-treated wines compared to the control non-treated wine [38].

Our samples showed brilliance values between 26.047 to 56.196 with a mean value of 41.886, and median 43.266. According to the literature, brilliance values range between 40 and 60 in young wines and are lower for older wines [24]. Indeed, only the six wine samples that showed hue values higher than one have brilliance values less than 40. There was found a strong negative correlation of vintage with hue (−0.733, significant at the 0.01 level) and positive (0.743 significant at the 0.01 level) with brilliance.

In general, there was observed that the proportion of red decreases with the ageing of wine while that of yellow increases. The blue proportion in all the samples did not surpass 13.72% whereas that of red and yellow showed values lower than 53.30 and 47.95%, respectively. The above values testify that the tested samples are rich in red and yellow hues. It seems that vintage was negatively correlated (0.683, significant at the 0.01 level) with the yellow proportion of the color and positively (0.728, significant at the 0.01 level) with the red. No correlation was observed with the blue proportion of the wine color. It is possible that other factors such as differences among the raw materials, the procedure used as well as the storage condition affect parameters such as pH, temperature and presence of free sulphur dioxide responsible for the formation of compounds related to red and blue hues of the final color of the wines [45,46]. Copigmentation processes and loss of monomeric anthocyanin due to the interaction with other wine compounds are responsible for the generation of other colored compounds during the ageing process [41].

Monitoring of the absorbance at 420 nm is usually used as a browning index in white wines. According to Fernandez-Zurbano et al., [47] browning of white dry wines falls in three categories; intense when absorbance was higher than 0.5, moderate between 0.2 and 0.5 and light when less than 0.2. Considering these categories, seven white Drama wines fall in the category of “moderate” browning whereas one in the “light” (Table 3). Oxidization, either enzymatic or chemical, of catechins and hydroxycinnamates that initially are colorless is responsible for the formation of yellow and brown products in white wines. According to, the observed variation (9.35 to 13.76) of the absorbance at 420 nm depends on the type of container used for the maturation.

Usually, the absorbance at 280 nm is related to the phenolics and is reported as the total phenolic index. The study of the white varieties showed that the absorbance at 280 nm varies from 3.767 to 4.066. In contrast with the high variation in the browning, there is not great variation observed among the phenolics in the tested samples.

### 3.3. Total Phenolics and Flavonoids Content and Antioxidant Activity of Wines

Numerous studies have shown that the geographical origin and grape varieties has a significant effect on the antioxidant capacity and the color of the wine. Total phenolic contents in our red wines vary from 0.666 to 3.291 mg/mL and in white from 0.226 to 0.527 mg/mL GAE (Table 4). The total phenolic contents was similar to that reported for Cretan white (0.349–0.352 mg/mL) and red (1.115–2.384 mg/mL) wines [48]. Moreover, our data for red wines are similar to those reported for commercial red wine samples of different origins [49,50]. This suggests that amateur wines could be compared to commercial wines regarding their phenolic contents. In general, the use of high doses of bentonite (overall negatively charged clay) reduces the total polyphenols, anthocyanins, tannins and polymeric pigments present in wines [16]. Taking into account the conclusions of Section 3.1, it seems that although the bentonite is used in high amounts as fining agent, the phenolic content is still comparable with that of commercial wines suggesting an initial load of phenolics in amateurs’ wines.

On the other hand, the levels of total flavonoids present in the tested samples vary from 0.304 to 1.667 mg/mL for the red and 0.064 to 0.340 mg/mL for the white wines. The values obtained for the red wines are higher than those reported for Romanian red wines [51]. In addition to the grape variety, the concentration of flavonoids in wine is strongly affected by the winemaking practices such as pressing and maceration that affect the degree of extraction from skins and especially from seeds which are rich in flavan-3-ol units [14].

It is generally recognized that phytochemicals have the ability to influence important cellular and molecular mechanisms related to health problems. In the present study, the antioxidant activity of home-made wines was assessed using three different antioxidant assays (ABTS, DPPH and FRAP). The antioxidant capacity of tested white wines differed significantly among wine samples as well as among the assays used (Table 4). The synergistic effect of different phenolics present in the wine as well as the presence of other bioactive constituents might justify the variation in the observed antioxidant activity.

While comparing different antioxidant assays, it is noticeable that the different assays differ in the antioxidant activity values reported for the same sample. The levels of TPC and TFC in red wines are significantly higher than those in white wines, and consequently, the antioxidant capacity of the wines differs significantly among the two types of wines. Indeed, depending on the used assay, the mean value of the antioxidant capacity of red wines was 4.25 to 5.29 times higher than that of the white wine counterparts.

Exposure to air and accompanying browning causes significant changes in the composition of phenolics present in the white wines, and this is measured with absorbance at 420 nm. Significant positive correlation (*p* < 0.05) was observed between the values of A420 nm and those of TFC (r = 0.712 and DPPH (r = 0.785) and negative correlation with ABTS (r = −0.800). This suggests that, on one hand, absorbance at 420 nm is mainly due to the variation in the flavonoid content and, on the other, that during oxidation of flavonoids, ABTS test is more sensitive to changes due to “browning”.

### 3.4. Exploratory Data Analysis of Wines

**Figure 1:**
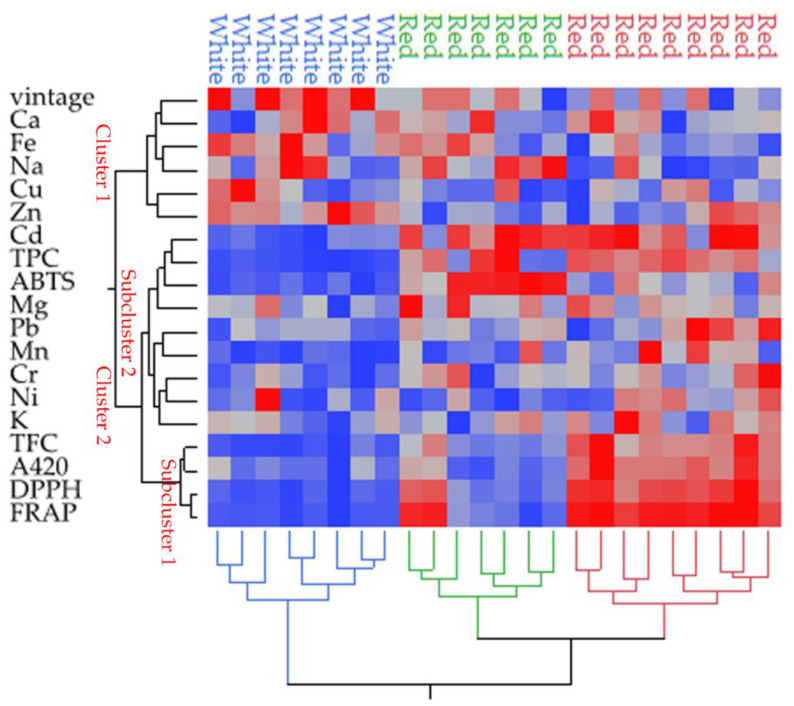
Dendrogram of two-way clustering of wines based on the variables measured in wines using Ward’s method on the standardized data to define distances between clusters. Blue areas in the map dendrogram indicate low values whereas the red areas indicate high values.

**Figure 2:**
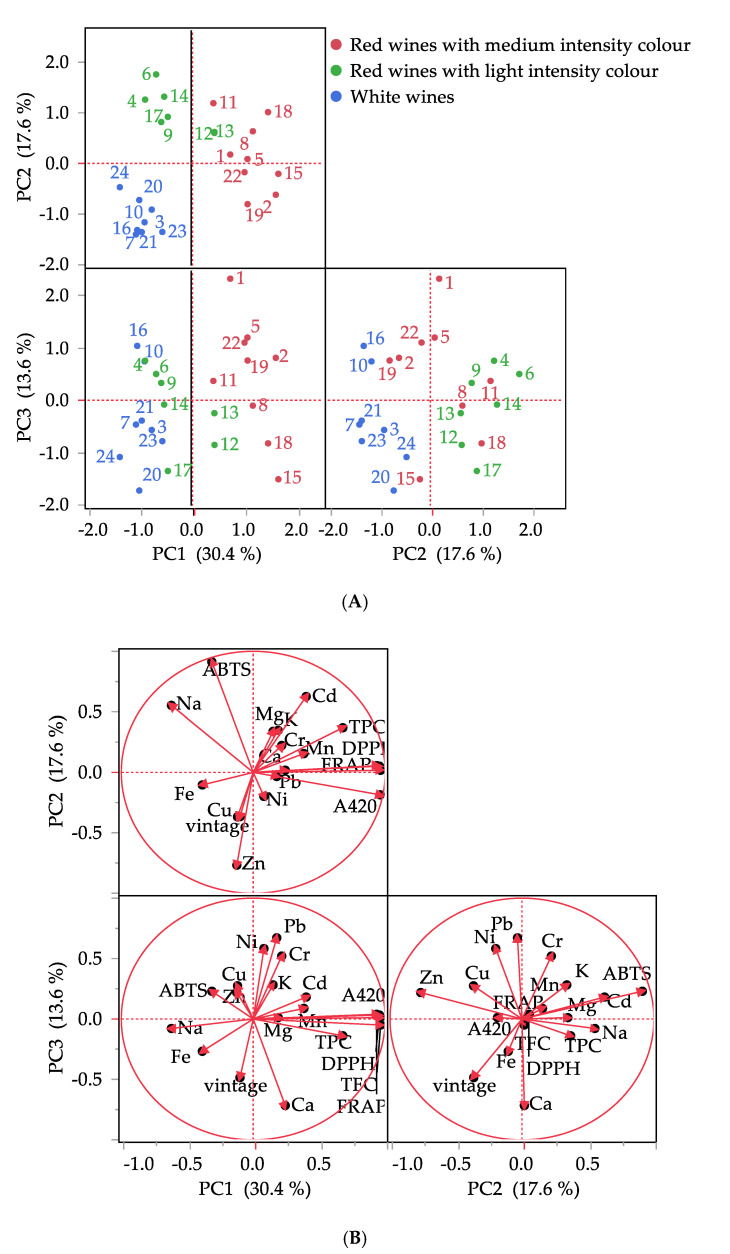
Principal component analysis (PCA). Biplot representation of samples (**A**) and variables (**B**) for wines. Different colors in the Biplot of samples represent the clusters as reported in the Figure 1.

To provide a certain classification for the studied wines made by amateurs, a heat map (Figure 1) was carried out, as well as PCA (Figure 2). Based on these results, the wines were divided into three groups. The white wines are depicted as a well-separated group whereas the red wines were classified in two sub-groups made of seven (noted with green color) and nine (noted with red color) samples, respectively.

The variables were separated in two groups (cluster 1 and cluster 2); cluster 1 comprises the elements Cu, Fe, Zn, Na, Ca and vintage, whereas the other is made of two subgroups, one of which (subcluster 1) contains TFC, DPPH, FRAP and absorption at 420 nm whereas the other (subcluster 2) comprises the rest of variables. Looking at cluster 1 where the Cu, Fe, Zn, Na and Ca elements are linked together reinforces the suggestion about interlinked variation of these elements possibly caused by the excess use of bentonite since this cluster cannot differentiate the wines.

As regarding the subcluster 1 where TFC, DPPH, FRAP and A420 are linked, in the previous sections, it was noted that variation in TFC was positively related to the DPPH and FRAP and that the absorbance at 420 nm in the white wines was linked with variation of flavonoid content and also “browning”. This group can differentiate the wines in two groups, all the white wines as well as the seven (green color cluster) of the red wines, showing lower values from the rest of the red wines (red color cluster).

In the heat map, one blue area in the region where all the white wines are located is distinguished, suggesting low values of variables of the cluster 2. Clustering of variables shows that ABTS is more related to the TPC than the other assays. In addition to ABTS and TPC, this group (subcluster 2) contains the rest of elements (Cd, Pb, Mg, Mn, Cr, Ni and K). This group can distinguish more clearly than the subcluster 1 the white wines (that have low values-blue areas) from the red wine samples. It is observed that in this subcluster Cd, Pb, Ni and Cr are linked, the presence of which is associated with cross-contamination from the equipment.

The PCA matrix consists of 24 samples and 19 variables. Based on the PCA, no anomalous sample (outlier) was present in the dataset. The seven first principal components (Eigenvalue >1) extracted explain 83.3% of the total variance. Bartlett’s test showed that all the variates were significant (*p* < 0.05), but we chose to plot only the first three. Principal component analysis reduced the dimensionality of the data set to three (Figure 2). The first principal component (PC1) explained 30.4% of the variance ratio, while PC2 explained an additional 17.6% with PC3 adding another 13.6% accounting for 61.6% of the total variance. Although 61.6% is not very a high value, a further increase of the number of components is not helpful in explaining the data. As expected, in the score plot in the plane defined by PC1 and PC2, the separation between white and red wines is evident. White wines (blue color) were located on the negative part of PC1 and PC2, and they were clearly separated from the red wines.

As expected, PCA distinguished the red from the white wines but at the same time discriminated the red wines in two groups. The red wines were positioned on the positive side as well as in the negative side of PC1. Results for red wines show that the two groups of red wine can be partially separated (9 red and 7 green (samples 4, 6, 9, 12, 13, 14 and 17) marked spots). Five (samples 4, 6, 9, 14 and 17) of the red samples of the green group are located in the negative part of the PC1 whereas two of them (12 and 13) on the positive part. The red group is located entirely on the positive part of the PC1. Looking more closely to the data, it was observed that the green group showed intensity values lower than 5.781, suggesting that all these wines are the wines considered of light intensity color whereas the wines of the red group are those considered with medium intensity color according to Iland, Ewart, Sitters, Markides and Bruer [42] (except from one (sample 8) with intensity 5.781, that is, however, close to the threshold value of 6).

The factors that contribute in the three-dimensional model are shown in Table 5 and the respective plots (Figure 2B). The first component (PC1) was best (factor loading > 0.5) described by attributes A420, FRAP, TFC, DPPH, TPC and Na. The second component (PC2) was characterized by attributes such as ABTS, Zn, Cd and Na. The third component (PC3) was characterized mainly by attributes such as Pb, Cr, Ni and Ca.

Regarding the variables, component 1 (PC1) is associated positively with most of the variables suggesting a partial effect of these variables on all the red wine samples with medium intensity color (red marked spots in Figure 2A). Na was negatively associated with PC1 and positively with PC2 suggesting a partial effect on the samples 4, 6, 9, 14 and 17 of the red wine group with light intensity color (green marked spots in Figure 2A). PCA was able to differentiate more the group of wines with medium intensity color; samples 8, 15 and 18 are grouped together in the positive axis of PC1 and the negative axis of PC3 (affected mainly from the attributes TFC, TPC Ca) whereas the rest of high intensity color samples are grouped in the positive axis of PC1 and the positive axis of PC3 (affected mainly from the attributes TFC, FRAP, DPPH, A420, Ni, Pb, Cr and Cd). PC2 and PC3 factors differentiated the red wines with light intensity color in the same way; samples 12, 13, 14 and 17 were located in the positive axis of PC2 and negative axis of PC3; the rest of the samples (4, 6 and 9) are located in the positive axis of PC2 and positive axis of PC3 being more affected by attributes Na, Ca, TPC and ABTS, Cr, Cd, respectively.

## 4. Conclusions

The results of the present study indicate that the wines produced by amateur winemakers of Drama region were safe since the level of toxic metals is far within the legal limits. Some micro- (Fe, Zn and Cu) and macroelements (K, Na, Ca and Mg) are present at lower levels of the ranges reported for commercial Greek and other international wines. On the other hand, although the data from the elemental analysis performed suggest the extensive use of bentonite by the amateur winemakers, the levels of phenolics and flavonoids are similar or even higher than those reported in the literature, making these wines not only safe but even richer in bioactive compounds. The red wines are distinguished by the red and blue hues whereas the white ones fall in the moderate light category suggesting that they have been properly stored. HCA and PCA analysis verified a clear separation of wines by type (red, white) as well as among the red wines by lightness and moderate intensity). Future studies are necessary to make a more in-depth evaluation of these wines monitoring the influence of other parameters (i.e., grape variety, soil composition, altitude, winemaking) on their quality. Although the big wine producers are those who have the expertise and the professional knowledge about quality wines, the present results showed that even the amateurs are able to produce wine that is not only safe but also that contains comparable or even higher phenolic compounds than the commercial wines reported in the literature. This could be mainly due to the grown public awareness of safety issues and the individual efforts of amateur winemakers. Thus, moderate consumption of these wines can be recommended since they can contribute to a healthy lifestyle.

## Figures and Tables

**Table 1:** Sample description of the tested wines from the region of Drama.

Sample	Type of Wine	Grape Variety	Vintage
S1	Red	Xinomavro/Merlot	2013
S2	Red	Merlot	2014
S3	White	Chardonnay/Muscat of Alexandria	2014
S4	Red	Merlot	2011
S5	Red	NR	2011
S6	Red	Merlot/Syrah	2014
S7	White	NR	2015
S8	Red	NR	2015
S9	Red	NR	2015
S10	White	Malagousia	2016
S11	Red	NR	2013
S12	Red	NR	2015
S13	Red	NR	2014
S14	Red	NR	2015
S15	Red	NR	2015
S16	White	Assyrtiko	2013
S17	Red	NR	2014
S18	Red	NR	2013
S19	Red	NR	2015
S20	White	Malagousia/Roditis/Assyrtiko	2016
S21	White	NR	2016
S22	Red	Limnio/Cabernet Sauvignon	2013
S23	White	NR	2016
S24	White	NR	2015

NR—data not available.

**Table 2:** Comparison of mean and median concentrations (as mg/L) of the elements determined in samples of red (*n* = 18) and white (*n* = 8) wines produced by amateur winemakers of Drama region in Greece *.

Element	OIV (International Organization of Vine and Wine) Maximum Levels (mg/L)	Mean (mg/L) ± Std. Deviation		Median (mg/L)		Minimum (mg/L)		Maximum (mg/L)	
		Red	White	Red	White	Red	White	Red	White
As (Arsenic)	0.2	<LOD ±	<LOD ±	<LOD		<LOD	<LOD	<LOD	<LOD
Cd (Cadmium)	0.01	0.004 ± 0.001	0.002 ± 0.000	0.005 a	0.002 b	0.003	0.002	0.005	0.003
Pb (Lead)	0.1	0.036 ± 0.015	0.025 ± 0.006	0.035 a	0.028 b	0.013	0.016	0.067	0.032
Cr (Chromium)	0.1	0.007 ± 0.005	0.003 ± 0.002	0.006 a	0.002 b	< LOD	< LOD	0.020	0.007
Cu (Copper)	1	0.050 ± 0.039	0.076 ± 0.065	0.038	0.051	0.010	0.016	0.144	0.206
Fe (Iron)	20	0.804 ± 0.325	1.103 ± 0.366	0.766	1.096	0.309	0.510	1.439	1.672
Mn (Manganese)	-	1.474 ± 1.262	0.503 ± 0.130	0.986 a	0.454 b	0.434	0.372	4.667	0.673
Ni (Nickel)	-	0.157 ± 0.098	0.156 ± 0.141	0.118	0.104	0.055	0.061	0.357	0.486
Zn (Zinc)	5	0.612 ± 0.212	0.900 ± 0.162	0.629 b	0.863 a	0.304	0.654	1.031	1.210
K (Potassium)	-	79.6 ± 18.4	70.1 ± 11.5	77.6	72.7	50.3	51.4	123.2	81.5
Na (Sodium)	80	2.7 ± 1.5	2.9 ± 1.5	2.7	2.7	0.8	1.4	5.2	5.3
Ca (Calcium)	-	6.1 ± 1.2	6.3 ± 1.7	6.2	6.4	3.7	3.7	8.4	8.8
Mg (Magnesium)	-	9.9 ± 1.1	8.9 ± 1.1	9.8	9.2	8.2	6.8	12.1	10.7

<LOD (Limit of Detection): Values lower that the limit of detection. * Within one line, medians of red and white wines with different letters differ significantly from each other (*p* < 0.05), as determined by the Independent-Samples Median Test.

**Table 3:** Color variables among the wines produced by amateur winemakers of Drama region in Greece *.

	Mean ± Std. Deviation	Median	Minimum	Maximum
*Red wine*				
Color Intensity	5.270 ± 2.237	5.897	1.411	9.159
Color Hue	0.922 ± 0.164	0.896	0.655	1.176
Brilliance (%)	41.886 ± 9.088	43.266	26.047	56.196
Proportion of yellow%	42.32 ± 4.20	42.28	34.54	47.95
Proportion of red%	46.56 ± 3.92	46.86	40.34	53.30
Proportion of blue%	11.13 ± 1.39	11.07	8.63	13.72
*White wine*				
A280 nm	3.935 ± 0.097	3.925	3.767	4.066
A420 nm	0.284 ± 0.135	0.236	0.081	0.502

* Data are averages of three independent measurements of each tested wine and are shown as mean ± SD values.

**Table 4:** Total phenolic and flavonoid concentrations as well as antioxidant capacity in wines by amateur winemakers of Drama region in Greece *.

	Mean (mg/mL) ± Std. Deviation		Median (mg/mL)		Minimum (mg/mL)		Maximum (mg/mL)	
	Red	White	Red	White	Red	White	Red	White
TPC	2.029 ± 0.776	0.363 ± 0.099	2.272 a	0.365 a	0.666	0.226	3.291	0.527
TFC	0.838 ± 0.430	0.156 ± 0.086	0.920 a	0.141 a	0.304	0.064	1.667	0.340
DPPH	3.500 ± 1.559	0.705 ± 0.279	4.184 a	0.737 a	0.843	0.270	5.438	1.023
ABTS	6.037 ± 3.330	1.142 ± 0.626	4.882 a	1.096 a	1.329	0.206	10.906	2.036
FRAP	5.238 ± 2.314	1.234 ± 0.268	6.623 a	1.367 a	0.901	0.722	7.068	1.419

* Within one line, medians of red and white wines with the same letter differ significantly from each other (*p* < 0.05), as determined by the Independent Samples Kruskal–Wallis test. TPC (Total Phenolic Content) are expressed as mg Gallic acid equivalents (GAE)/mL, TFC (Total Flavonoid Content) as mg Catechin equivalents (CATE)/mL whereas DPPH (2,2-diphenyl-1-picrylhydrazyl), ABTS (2,2′-azinobis (3-ethylbenzothiazoline-6-sulfonic acid) and FRAP (Ferric reducing antioxidant power) as mg Trolox equivalent (TE)/mL.

**Table 5:** Rotated factor loadings.

	Component 1	Component 2	Component 3
vintage	−0.100600	−0.365712	**−0.484852**
Cd	0.401070	**0.625847**	0.181428
Pb	0.178149	−0.033854	**0.671428**
Cr	0.215748	0.222721	**0.520405**
Cu	−0.119180	**−0.367322**	0.274617
Fe	**−0.386043**	−0.103756	−0.267366
Mn	**0.383935**	0.155633	0.087432
Ni	0.080965	−0.198611	**0.582837**
Zn	−0.124327	**−0.767032**	0.218482
K	0.150334	0.339505	0.281856
Na	**−0.619868**	**0.552884**	−0.078169
Ca	0.242568	0.018137	**−0.715440**
Mg	0.189191	0.347689	0.011345
TPC	**0.678959**	0.369308	−0.139029
TFC	**0.965318**	0.017392	−0.049698
DPPH	**0.956747**	0.051815	0.032724
ABTS	−0.313627	**0.912607**	0.229632
FRAP	**0.935341**	0.056037	0.038930
A420	**0.962183**	−0.185351	0.011776

Bold numbers note the values that are of interest and based on them was made the discussion.

## Abbreviations

(S)-(-)-6-hydroxy-2,5,7,8-tetramethylchroman-2-carboxylic acid (Trolox); 2,2′-azinobis (3-ethylbenzothiazoline-6-sulfonic acid (ABTS); 2,2-diphenyl-1-picrylhydrazyl (DPPH); 2,4,6-tripyridyl-s-triazine (TPTZ); Catechin equivalents (CATE); Coefficient of variation (CV); Ferric reducing antioxidant power (FRAP); Gallic acid equivalents (GAE); Hierarchical Cluster Analysis (HCA); High-Performance Liquid Chromatography (HPLC); Inductively Coupled Plasma Optical Emission Spectrometer (ICP-OES); International Organization of Vine and Wine (OIV); International Union of Pure and Applied Chemistry (IUPAC); Limit of detection (LOD); Limit of the quantification (LOQ); Principal Component Analysis (PCA); Protected Geographical Indication (PGI); Relative Standard Deviation of Repeatability (RSD); Standard deviation (SD); Total flavonoid content (TFC); Total phenolic content (TPC); Trolox equivalents (TE); Wine color hue (T); Wine color intensity (I).
